# Supplementary material for: Horizontally transferred genes in the ctenophore Mnemiopsis leidyi
Source: PeerJ. 2018 Jun 15;6:e5067. doi: 10.7717/peerj.5067 (PMC6005172; doi:10.7717/peerj.5067)
Supplement: Supplemental Information 1 [file peerj-06-5067-s001.pdf]

Supplementary text: commands and parameters for phylogenetic analyses

All files and scripts are available at: https://github.com/josephryan/2018-Hernandez_and_Ryan_HGT

1. Alignments

Alignments were generated using MAFFT version 7.305 and trimmed with Gblockswrapper version 0.91b using the following commands:

```
mafft seqs.fa > aln.fa
```

```
Gblockswrapper aln.fa
```

Gblockswrapper is available here:
https://bitbucket.org/caseywdunn/labcode/src/master/scripts_phylogenomics_21Feb2009/Gblockswrapper

```
perl -pi -e 's/ //g' aln.fa-gb
```

```
fasta2phy.pl aln.fa-gb > aln.fa-gb.phy
```

fasta2phy.pl is a script bundled with JFR-PerlModules (Release 1.0+)
https://github.com/josephryan/JFR-PerlModules/

2. Tree inference

We performed maximum likelihood analyses on the alignments using RAxML version 8.1.21:

```
raxmlHPC -p 1234 -m PROTGAMMAGTR -n ALN -s aln.fa-gb.phy
```

3. Hypothesis testing

All commands used for hypothesis testing are in the script test_hypothesized_phylogenies uploaded to GitHub. We used the SOWH test as implemented in SOWHAT version 0.36 and the AU test implemented in CONSEL version 0.20. The script test_hypothesized_phylogenies generates the metazoan constraint tree as well as bootstrap trees to test as alternative hypotheses against the best tree (i.e., tree indicating HGT) produced by RAxML. The following command is used run the script:

```
perl test_hypothesized_phylogenies.pl aln.fa-gb.phy dir/
```

4. Single copies of bootstrap trees

To make sure that each bootstrap tree only appeared once in a set of 100 generated bootstrap trees (i.e., suboptimal trees) we used the ape package in R with the following commands:

```
library (ape)

bs<-read.tree(file='RAxML_bootstrap.ALN.bs')

unique.multiPhylo(bs)
```

5. Manually generated suboptimal trees

We used suboptimal trees to address the problem of selection bias which occurs when using the AU test. We manually created suboptimal trees in order to get a feel for how different bootstrap trees were from the optimal tree. Suboptimal trees were manually created by switching the positions of taxa in clades consisting of three taxa from the best tree (Fig. S2). We included 10 manually generated suboptimal trees, or in cases with less than 10 trees, as many as could be generated in each analysis for the AU test. To optimize manually generated suboptimal trees we performed maximum likelihood analyses before implementing the AU test. All manually created suboptimal trees have been uploaded to the accompanying GitHub site.

We used the following commands to optimize each manually generated suboptimal tree:

```
raxmlHPC -p 1234 -m PROTGAMMAGTR -n suboptree1 -s aln.fa-gb.phy -g
RAxMLbestTree1_equalbranch.ALN
```

To run the AU test implemented through CONSEL on the best, metazoan constraint, and manually generated suboptimal trees*:*

```
cat RAxML_bestTree.ALN RAxML_bestTree.metatree RAxML_bestTree.suboptree1
RAxML_bestTree.suboptree2 RAxML_bestTree.suboptree3
RAxML_bestTree.suboptree4 RAxML_bestTree.suboptree5
RAxML_bestTree.suboptree6 RAxML_bestTree.suboptree7
RAxML_bestTree.suboptree8 RAxML_bestTree.suboptree9
RAxML_bestTree.suboptree10 >> 12trees.tre

raxmlHPC -f g -m PROTGAMMAGTR -n 12trees -s aln.fa-gb.phy -z 12trees.tre

seqmt --puzzle RAxML_perSiteLLs.12trees

makermt RAxML_perSiteLLs
```

```
consel RAxML_perSiteLLs

catpv RAxML_perSiteLLs
```

6. Violin plots

We used violin plots to visualize comparisons of likelihood scores between the best tree (i.e., tree indicating HGT), metazoan constraint tree, and suboptimal trees. Violin plots were generated using the script likelihood_violins available on the accompanying GitHub site. We also made comparisons between the tree space covered by using bootstrap trees as suboptimal trees (Fig. 3) versus manually created suboptimal trees in the AU test (Fig. S3). We found that the bootstrap trees covered a wider range of tree space than the manually created trees. Therefore, bootstrap trees provide a more stringent test.

7. HMMER analysis on HGT candidates absent in other animals

To ensure that these genes (ML012034a, ML18354a, ML219316a) are truly absent in other animals and are not an artifact caused by long-branch *M. leidyi* sequences, we performed HMMER searches as an additional method to detect homologous sequences in animals. We used the EMBL-EBI HMMER interface to search for homologous animal sequences in the UniProtKB database. ML012034a had significant hits (E-value ≤ 0.1) to two animals (*Macrostomum lignano* and *Folsomia candida*). If these were not contaminants, we would expect to find this gene more widely represented in Metazoa. ML18354a and ML219316a each had hits to a single animal, *Lygus Hesperus* and *Philodina roseola* respectively. These also are likely to be contaminants since they lack representation among a wider range of animals. In Table S5 we show the best non-animal and animal E-values for each of these genes, as well as the species for the best hits.

8. Bayesian inference on *bona fide* HGTs

We used Bayesian methods to validate the topologies resulting from maximum likelihood analyses. We used MrBayes version 3.2.6 under a GTR model to generate Bayesian trees on six of the nine *bona fide* HGTs (ML00955a, ML00555a, ML005129a, ML02771a, ML49231a, ML42441a); the remaining three lacked hits to other animals. We found no major differences between the resulting gene trees and the original maximum likelihood trees. All NEXUS files and embedded commands have been uploaded to the accompanying GitHub in the directory 07-BAYES_INFERENCE.

9. Maximum likelihood analysis with broader taxon sampling of ctenophores for *bona fide* HGTs

To test if including sequences from additional ctenophores affect the outcome of our phylogenetic analyses, we performed maximum likelihood analyses with broader taxon sampling for six of the nine *bona fide* HGTs (ML00955a, ML00555a, ML005129a, ML02771a, ML49231a, ML42441a) which had BLASTP hits to other animals. We used the original sequences from the alignments for maximum likelihood analyses, but also included homologous sequences identified in other ctenophores (Fig 4). All sequences were aligned in MAFFT and trimmed with Gblockswrapper. We then performed maximum likelihood analyses using RAxML and the GTR model. We found no major differences in our resulting phylogenetic topologies compared to our original gene trees, since these HGTs appear to have occurred early in ctenophore evolution. All results have been uploaded to the GitHub repository in the directory 08-MAX_LIKELIHOOD_CTENO.

10. Maximum likelihood analysis with broader protistan taxon sampling

RefSeq includes only a limited number of available non-metazoan eukaryotic lineages, and while we find the loss of a gene from Filasterea, the stem of Choanoflagellatea, the stem of Porifera plus Parahoxozoa, but not Ctenophora a more complex scenario than a horizontal gene transfer, it is important to bring as much evidence on this question as is available. As such, we have expanded our protistan taxonomic sampling in the phylogenetic analyses of the nine bona fide HGTs. We performed HMMER searches against the Ensembl Protists database to collect the top five similar sequences from this database that were not included in the original analyses. We added these sequences to the original RefSeq-based datasets from the main paper and repeated the phylogenetic analyses as outlined in the methods. All resulting treefiles and alignments have been uploaded to the GitHub repository in the directory 09-EXPANDED_EUK_MAXLIKELIHOOD.

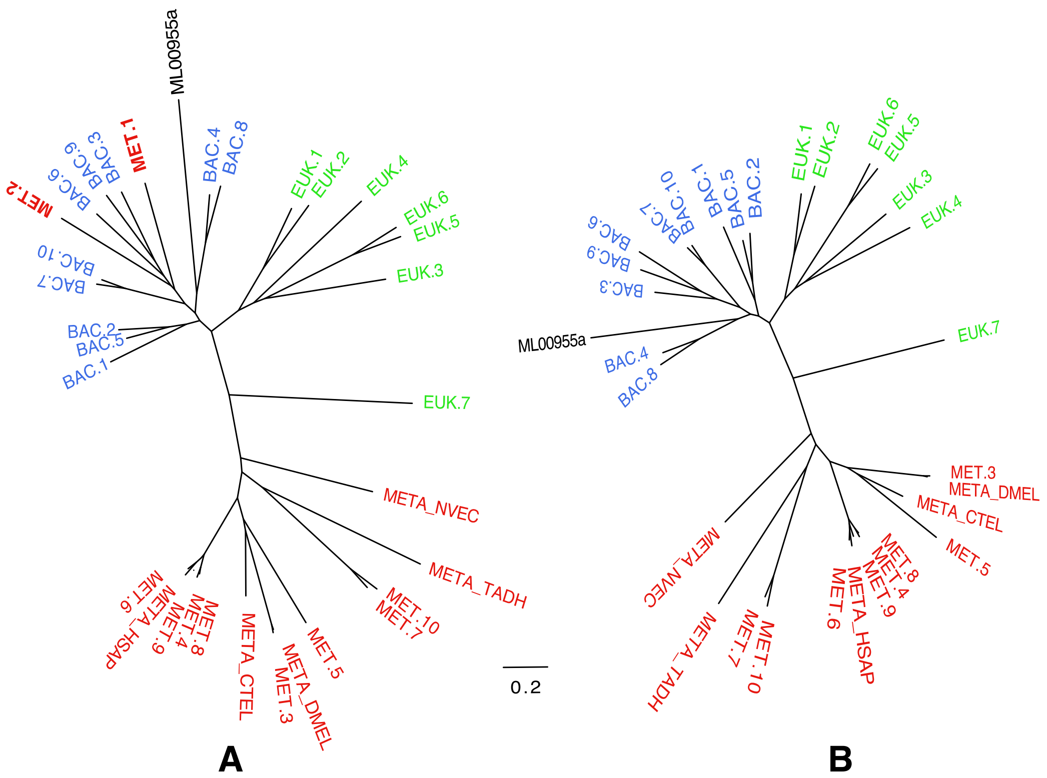

Figure S1. Maximum-likelihood analyses on an HGT candidate that includes metazoan sequences outside of the main metazoan clade. ML00955a (in black) is the *M. leidyi* HGT candidate. (A) Because there were only two non-*Mnemiopsis* animal sequences outside of the main animal clade (i.e, MET.1 and MET.2), these sequences were considered potential contaminants and removed. (B) RAxML analysis on the same alignment after pruning MET.1 (*Pantholops hodgsonii*) and MET.2 (*Caenorhabditis remanei*). Taxa that are prefixed "META_" are from our alien_index database version 0.01 (i.e., META_NVEC (*Nematostella vectensis*), META_TADH (*Trichoplax adhaerens*), META_HSAP (*Homo sapiens*), META_CTEL (*Capitella teleta*), META_DMEL (*Drosophila melanogaster*). MET=Metazoa; BAC=Bacteria; EUK=Eukaryota; FUN=Fungi; More details for each taxa are specified in Table S3.

**Species**	**Lineage**
Acanthamoeba castellanii	Amoebozoa
Aspergillus nidulans	Fungi
Candidatus aquiluna sp imcc13023	Bacteria / Terrabacteria
Candidatus nitrosopumilus salaria bd31	Archaea
Candidatus pelagibacter sp imcc9063	Bacteria / Proteobacteria
Glaciecola pallidula dsm 14239 acam 615	Bacteria / Proteobacteria
Marine gamma proteobacterium htcc2080	Bacteria / Proteobacteria
Marine group i thaumarchaeote scgc aaa799 b03	Archaea
Marinobacter adhaerens hp15	Bacteria / Proteobacteria
Phaeodactylum tricornutum	Stramenopiles
Saccharomyces cerevisiae	Fungi
Thalassiosira pseudonana	Stramenopiles
Amphimedon queenslandica	Animal / Porifera
Capitella teleta	Animal / Annelida
Crassostrea gigas	Animal / Mollusca
Daphnia pulex	Animal / Arthropoda
Drosophila melanogaster	Animal / Arthropoda
Helobdella robusta	Animal / Annelida
Lottia gigantea	Animal / Mollusca
Nematostella vectensis	Animal / Cnidaria
Strigamia maritima	Animal / Arthropoda
Strongylocentrotus purpuratus	Animal / Echinodermata
Trichoplax adhaerens	Animal / Placozoa
Homo sapiens	Animal/ Chordata

Table S1. **Species used in initial alien_index run to identify HGT candidates.** This database is available here: http://ryanlab.whitney.ufl.edu/downloads/alien_index/

Gene ID	AI	Best E-Value	Best non-alien E-value
ML070218a	240.2673574	9.00E-117	2.00E-12
ML21002a	169.1873241	3.00E-91	9.00E-18
ML018031a	163.5889021	9.00E-72	no hits
ML132017a	161.5864216	2.00E-117	3.00E-47
ML120721a	156.2393141	7.00E-107	5.00E-39
ML012034a	151.0543254	1.00E-69	4.00E-04
ML00955a	143.6765665	8.00E-145	2.00E-82
ML046416a	133.5499354	1.00E-91	1.00E-33
ML00555a	132.8567882	4.00E-79	2.00E-21
ML005129a	132.163641	4.00E-65	1.00E-07
ML02771a	131.9404975	5.00E-58	no hits
ML06718a	124.339595	1.00E-54	no hits
ML296211a	117.0953675	7.00E-74	5.00E-23
ML03277a	116.0455454	4.00E-51	no hits
ML085726a	107.1228871	9.00E-84	3.00E-37
ML02232a	105.9189143	1.00E-46	no hits
ML49231a	98.09486827	2.00E-58	8.00E-16
ML102910a	87.60359405	9.00E-39	no hits
ML073257a	85.60111355	2.00E-55	3.00E-18
ML18354a	85.41879199	8.00E-38	no hits
ML019144a	84.50250126	6.00E-46	3.00E-09
ML177319a	83.58621053	1.00E-85	2.00E-49
ML227811a	82.09455565	2.00E-82	9.00E-47
ML049014a	79.89733107	2.00E-41	1.00E-06
ML207910a	79.38650545	1.00E-74	3.00E-40
ML1541114a	76.67845525	1.00E-117	2.00E-84
ML092610a	70.6869907	2.00E-45	1.00E-14
ML03547a	69.77069997	4.00E-44	8.00E-14
ML22167a	66.08182052	1.00E-64	5.00E-36
ML102221a	65.85867696	1.00E-88	4.00E-60
ML009115a	55.95518941	1.00E-33	2.00E-09
ML00881a	55.26204223	1.00E-24	no hits
ML00882a	51.57316278	4.00E-23	no hits
ML00556a	50.94455412	3.00E-35	4.00E-13
ML42441a	50.65687205	2.00E-130	2.00E-108
ML23958a	48.64196903	3.00E-38	4.00E-17
ML219316a	46.05170186	1.00E-20	no hits

Table S2. HGT candidates identified by alien index (Fig. 1A). AI designates the alien index values for each candidate. All HGT candidates had a better hit to a non-animal (shown under the best E-value). Each of these genes were BLASTed against the RefSeq database (Fig.1B). Genes that are not highlighted show HGT candidates

with no significant BLAST hits to animals in the RefSeq database. Genes highlighted in orange underwent maximum likelihood analyses, but were not supported as HGTs (i.e., they were closely related to animals). Genes highlighted in blue show HGT candidates that were more closely related to non-animals and underwent hypothesis testing (Fig. 1C).

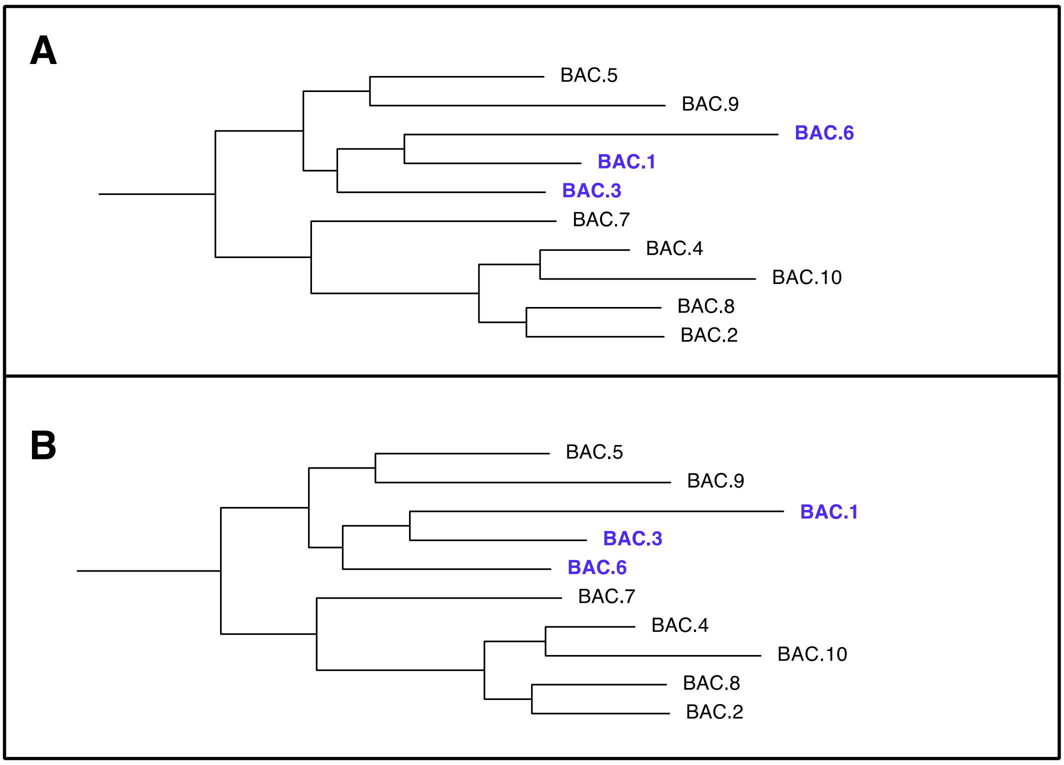

Figure S2. Examples of manually generated suboptimal trees. Suboptimal trees were manually generated by taking the best RAxML tree from each alignment and switching the positions of taxa in clades of three. All branch lengths were made equal when performing taxa switches and then optimized using RAxML. Blue text in the figure highlights the clade of three taxa in which positions of taxa will be rearranged. (A) Clade of bacteria resulting from the best maximum-likelihood tree. (B) Manually generated suboptimal tree that resulted from rearrangement of taxa highlighted in blue from the clade in (A).

Label	Species	Accession no.
Bac. 1	*Solitalea canadensis*	WP_014679673.1
Bac. 2	*Pontibacter actiniarum*	WP_025607756.1
Bac. 3	*Microbulbifer agarilyticus*	WP_010132679.1
Bac. 4	*Stenotrophomonas*	WP_055768138.1
Bac. 5	*Rufibacter* sp. DG31D	WP_053093794.1
Bac. 6	*Idiomarina zobellii*	WP_053954579.1
Bac. 7	*Echinicola vietnamensis*	WP_015263982.1
Bac. 8	*Arenimonas metalli*	WP_052575499.1
Bac. 9	*Arsukibacterium* sp. MJ3	WP_046552654.1
Bac. 10	*Anditalea andensis*	WP_035071114.1
Euk. 1	*Thalassiosira pseudonana* CCMP1335	XP_002296777.1
Euk. 2	*Guillardia theta* CCMP2712	XP_005831049.1
Euk. 3	*Micromonas* sp. RCC299	XP_002501910.1
Euk. 4	*Chondrus crispus*	XP_005713402.1
Euk. 5	*Saprolegnia parasitica*CBS 223.65	XP_012200393.1
Euk. 6	*Aphanomyces invadans*	XP_008879597.1
Euk. 7	*Galdieria sulphuraria*	XP_005705561.1
Met. 3	*Drosophila willistoni*	XP_015033401.1
Met. 4	*Protobothrops mucrosquamatus*	XP_015682900.1
Met. 5	*Helobdella robusta*	XP_009014349.1
Met. 6	*Serinus canaria*	XP_009093895.1
Met. 7	*Apis florea*	XP_012343105.1
Met. 8	*Python bivittatus*	XP_007437127.1, XP_007437128.1, XP_007437129.1
Met. 9	*Gekko japonicus*	XP_015268057.1
Met. 10	*Halyomorpha halys*	XP_014272262.1

Table 3A. Taxa details from Figure 2(A), (B), and Figure S1. Labels indicate the taxa labels in Figures 2(A), 2(B), and S1. Each label specifies the species name and accession number downloaded from RefSeq.

Label	Species	Accession no.
Bac. 1	*Bordetella* sp. N	WP_057653136.1
Bac. 2	*Lysobacter* sp. Root690	WP_056114344.1
Bac. 3	*Amycolatopsis methanolica*	WP_017981605.1
Bac. 4	*Achromobacter xylosoxidans*	WP_013396304.1
Bac. 5	*Knoellia flava*	WP_052116956.1
Bac. 6	*Nocardioides* sp. Soil774	WP_056601935.1
Bac. 7	*Actinosynnema mirum*	WP_012783612.1
Bac. 8	*Arthrobacter* sp. MA-N2	WP_028266024.1
Bac. 9	*Curtobacterium* sp. Leaf261	WP_055954596.1
Bac. 10	*Phycicoccus* sp. Soil803	WP_057377348.1
Euk. 1	*Cucumis melo*	XP_008448951.1
Euk. 2	*Medicago truncatula*	XP_003611170.1
Euk. 3	*Glycine max*	XP_003517450.1
Euk. 4	*Tarenaya hassleriana*	XP_010540447.1
Euk. 5	*Beta vulgaris* subsp. *vulgaris*	XP_010678653.1
Euk. 6	*Musa acuminata* subsp. *malaccensis*	XP_009398075.1
Euk. 7	*Cicer arietinum*	XP_004510892.1
Euk. 8	*Vigna radiata* var. *radiata*	XP_014521222.1
Euk. 9	*Morus notabilis*	XP_010108055.1
Euk. 10	*Solanum tuberosum*	XP_015169639.1
Fun. 1	*Encephalitozoon cuniculi* GB-M1	NP_586424.1
Fun. 2	*Torulaspora delbrueckii*	XP_003680922.1
Fun. 3	*Trametes versicolor* FP-101664 SS1	XP_008044753.1
Fun. 4	*Kazachstania africana* CBS 2517	XP_003954912.1
Fun. 5	*Dichomitus squalens* LYAD-421 SS1	XP_007365208.1
Fun. 6	*Puccinia graminis* f. sp. *tritici* CRL 75-36-700-3	XP_003322083.2
Fun. 7	*Coniophora puteana* RWD-64-598 SS2	XP_007775552.1
Fun. 8	*Eremothecium cymbalariae* DBVPG#7215	XP_003647557.1
Fun. 9	*Moniliophthora roreri* MCA 2997	XP_007853138.1
Fun. 10	*Encephalitozoon intestinalis* ATCC 50506	XP_003073966.1
Met. 1	*Jaculus jaculus*	XP_004666971.1
Met. 2	*Chrysochloris asiatica*	XP_006863382.1
Met. 3	*Odobenus rosmarus divergens*	XP_004398631.1
Met. 4	*Priapulus caudatus*	XP_014664176.1
Met. 5	*Ovis aries musimon*	XP_011978134.1
Met. 6	*Trichechus manatus latirostris*	XP_004376138.1
Met. 7	*Dasypus novemcinctus*	XP_004447311.1
Met. 8	*Nannospalax galili*	XP_008854136.1, XP_008854137.1, XP_008854138.1
Met. 9	*Rattus norvegicus*	XP_008766000.1
Met. 10	*Capra hircus*	XP_005693518.1

Table S3B. Taxa details from Figure 2(C), (D). Labels indicate the taxa labels in Figures 2(C) and 2(D). Each label specifies the species name and accession number downloaded from RefSeq.

Label	Species	Accession no.
Bac. 1	*Leptospira meyeri*	WP_004787080.1
Bac. 2	*Nonomuraea coxensis*	WP_026214713.1
Bac. 3	*Actinomadura rifamycini*	WP_051300306.1
Bac. 4	*Marmoricola aequoreus*	WP_030484673.1
Bac. 5	*Kytococcus sedentarius*	WP_049758670.1
Bac. 6	*Nocardioides*	WP_056707204.1
Bac. 7	*Myxococcus fulvus*	WP_046713442.1
Bac. 8	*Chondromyces apiculatus*	WP_044234766.1
Bac. 9	*Phycicoccus* sp. Root101	WP_056918311.1
Bac. 10	*Nitriliruptor alkaliphilus*	WP_052668139.1
Euk. 1	*Acanthamoeba castellanii* str. Neff	XP_004367908.1
Euk. 2	*Capsaspora owczarzaki* ATCC 30864	XP_004343108.1
Euk. 3	*Aphanomyces invadans*	XP_008862617.1
Euk. 4	*Saprolegnia diclina* VS20	XP_008605694.1
Euk. 5	*Acytostelium subglobosum* LB1	XP_012754808.1
Euk. 6	*Monosiga brevicollis* MX1	XP_001745020.1
Euk. 7	*Dictyostelium discoideum* AX4	XP_645447.1
Euk. 8	*Aureococcus anophagefferens*	XP_009039197.1
Euk. 9	*Monoraphidium neglectum*	XP_013896596.1
Euk. 10	*Fonticula alba*	XP_009493401.1
Fun. 1	*Punctularia strigosozonata*	XP_007381255.1
Fun. 2	*Wallemia ichthyophaga* EXF-994	XP_009266318.1
Fun. 3	*Coniophora puteana* RWD-64-598 SS2	XP_007766588.1
Fun. 4	*Puccinia graminis* f. sp. tritici CRL 75-36-700-3	XP_003327856.1
Met. 2	*Amyelois transitella*	XP_013199717.1
Met. 3	*Plutella xylostella*	XP_011563596.1
Met. 5	*Papilio polytes*	XP_013143494.1
Met. 6	*Bombyx mori*	XP_012546269.1

Table S3C. Taxa details from Figure 2(E), (F). Labels indicate the taxa labels in Figures 2(E) and 2(F). Each label specifies the species name and accession number downloaded from RefSeq.

*Note: All downloaded RefSeq sequences used for the analyses have been uploaded to the accompanying GitHub site.

Genes	% Identity							
	E. dunlapae	*C. astericola*	*V. multiformis*	*P. bachei*	*D. glandiformis*	*B. abyssicola*	*B. infundibulum*	*M. leidyi* (FL)
ML012034a	59	57	58	58	64	63	65	60
ML005129a	62	68	99	75	61	55	73	99
ML18354a	88	90	89	90		31	92	95
ML00955a	61	68	65	62	70		79	99
ML02771a		48	54	43	58		34	100
ML49231a	56			67	71	72		99
ML00555a	41	70		63		69		91
ML42441a		65	69	26			80	100
ML219316a		51	58	54	64	75	90	97

Table S4. Percent identity from BLAST for genes from Figure 4.

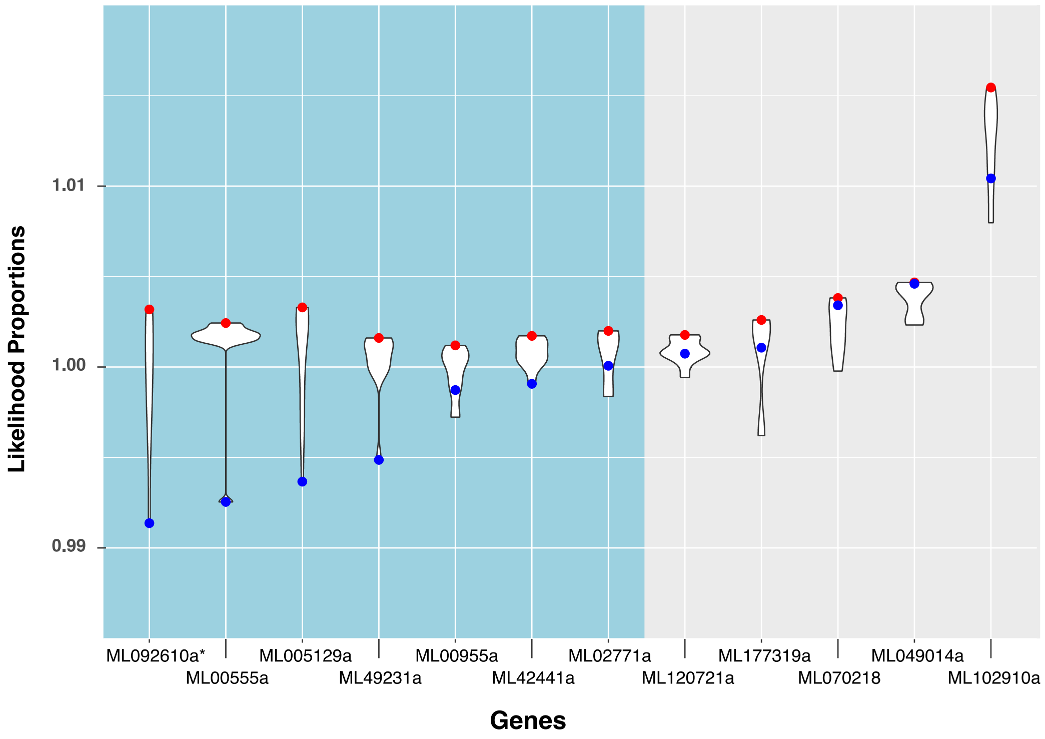

Figure S3. Manually generated suboptimal trees. Suboptimal distributions are a result of manually shuffling clades of three taxa. Results from analyses using bootstrap trees (Fig. 3) and manually created suboptimal trees are similar, but the spread of distribution of bootstrap trees is greater, making conclusions derived from comparisons to the bootstrap distributions more conservative. Red points indicate likelihood proportions of the best tree (i.e., tree indicating HGT). Blue points indicate likelihood proportions of the metazoan constrained tree (i.e., tree contradicting HGT). The side in teal shows HGT candidates validated by hypothesis testing and the side in gray shows HGT candidates unsupported by hypothesis testing. The asterisk indicates a gene that was later removed from contention.

Gene	Best non-animal E-val	Non-animal species	Best animal E-val	Animal species
ML012034a	5.60E-77	Pythium ultimum (Euk)	3.60E-07	Macrostomum lignano
ML18354a	2.80E-76	Oryza rufipogon (Euk)	2.00E-20	Lygus hesperus
ML219316a	6.70E-43	Gimesia maris DSM 8797 (Bac)	1.60E-17	Philodina roseola

Table S5. HMMER analysis on genes classified as absent in animals in RefSeq. Non-animals species indicate the best hit to non-animals, while animal species indicate the best animal hits.
